# Pyriform Turbinoplasty and Lateral Nasal Wall Lateralization: Practical Hints on How We Do It

**DOI:** 10.7759/cureus.32584

**Published:** 2022-12-16

**Authors:** André De Sousa Machado, Hans R Briner, Daniel Simmen

**Affiliations:** 1 Otolaryngology - Head and Neck Surgery, Centro Hospitalar Universitário do Porto, Porto, PRT; 2 Faculty of Health Sciences, University of Beira Interior, Covilhã, PRT; 3 Otolaryngology, Hirslanden Klinik, Zurich, CHE

**Keywords:** chronic rhinosinusitis, functional endoscopic sinus surgery (fess), ear nose and throat/otolaryngology, lateral nasal wall, turbinates

## Abstract

Pyriform turbinoplasty (PT) is a surgical option for the management of turbinate hypertrophy. The philosophy and goal of the procedure are to improve the symptoms of a restricted airway while preserving function. We report a case of surgical management of inferior turbinate (IT) hypertrophy with PT and lateral nasal wall lateralization (LNWL). PT and LNWL improve nasal airflow, providing a wider nasal cavity by the removal of the bone of the IT. Sustained symptomatic improvement has been documented and is less susceptible to the influence of turbinate hypertrophy with other techniques.

## Introduction

The external nasal valve area comprises the septum medially, superiorly and laterally the caudal margin of the upper lateral cartilages. The head of the inferior turbinate (IT) and the floor of the pyriform aperture are also part of this region, influencing the respective nasal airflow [1]. The nasal valve area is the narrowest conduit of airflow in the nose and its resistance is influenced by the size of the IT. When the response to medical treatment is poor, surgical management of IT becomes an option. Nasal valve surgery can either strengthen the lateral nasal wall with grafting techniques or increase its cross-sectional area with septoplasty or turbinoplasty [1]. Simmen and Jones described pyriform turbinoplasty (PT) as an option for the management of turbinate hypertrophy [1]. The philosophy and goal of the procedure are to improve the symptoms of a restricted airway while preserving function. These goals are achieved by improving the airway without excessive trimming of the turbinates which can result in a reduced sensation of airflow, dryness, and crusting along with an unphysiological distribution of airflow [2,3].

## Technical report

Indications for surgery

Hypertrophy of the IT that is unresponsive to medical treatment, such as topical corticosteroids and nasal douching.

Patient preparation and anesthetic technique

Although this technique can be performed under local anesthesia, we consider pertinent the use of general anesthesia to free the surgeon from some discomfort that the patient might feel and to better manage any possible bleeding by prompt suction, Moreover, the patient remains unaware of unpleasant sensation which can be an advantage when performing procedures in the nasal mucosa. A topical vasoconstrictor (such as xylometazoline) can be administered 20 minutes prior to surgery. A key point regarding general anesthesia is to ensure bradycardia. Oral metoprolol can be administered the day before the surgery when there are no contraindications, for example, asthma. Induction with a laryngeal mask produces less stimulation and is now our main method of protecting the airway, producing less stimulation on extubation, less coughing, and fewer chances of bleeding when the patient is waking up in recovery. Further, positioning the patient’s body at 20 degrees up reduces the venous pressure.

We report a case of surgical management of IT hypertrophy with PT and lateral nasal wall lateralization (LNWL) (Video 1).

**Video 1:** PT and LNWL performed step-by-step. PT = pyriform turbinoplasty; LNWL = lateral nasal wall lateralization

Pyriform turbinoplasty

**Figure 1:**
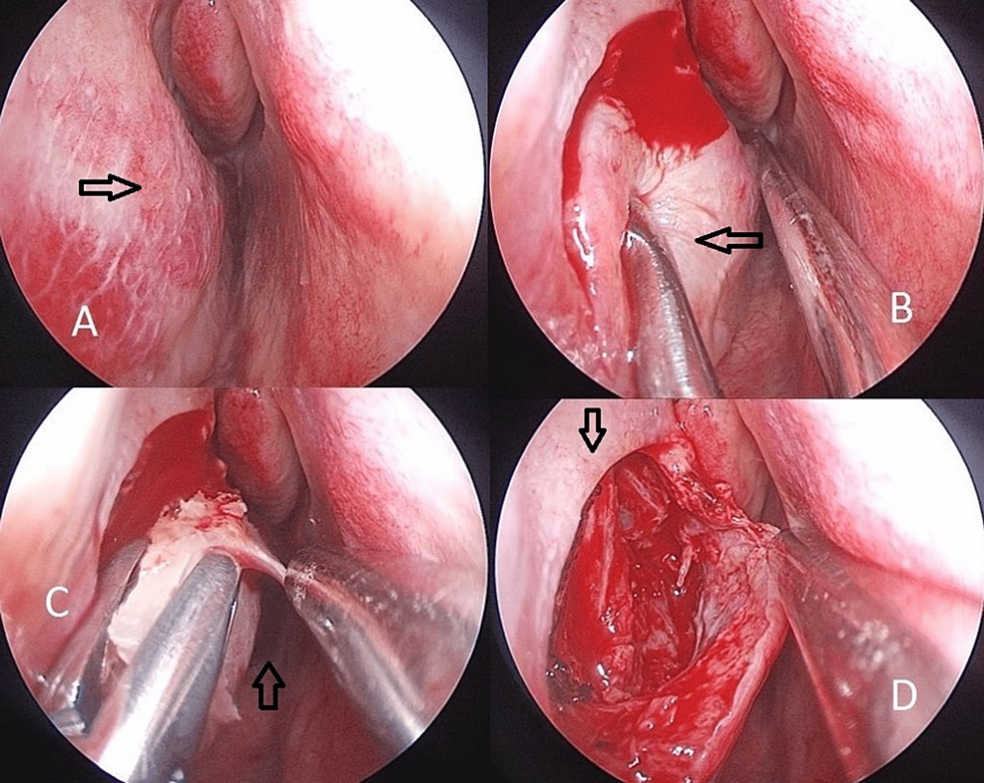
PT performed step-by-step. A: Preoperative view of the shoulder of the inferior turbinate. B: Horizontal incision made over the base of the inferior turbinate. C: Mucosal flap raised to expose the attachment of the inferior turbinate bone into the lateral wall. Removal of the base of the shoulder of the inferior turbinate after osteotomy. D: Appearance after removal of the shoulder of the inferior turbinate. PT = pyriform turbinoplasty

The “shoulder” of the IT, composed of the maxilla’s frontal process and the respective articulation with a portion of the lacrimal bone, is more difficult to lateralize compared with its middle and posterior parts. A horizontal incision is made over the base of the IT where its “shoulder” is located. A flap is raised from below, extended superiorly to reveal the attachment of the bone of the IT onto the lateral nasal wall. An osteotome can be used and placed at the inferior portion of the “shoulder” of the IT that intrudes into the nasal valve area. The lacrimal duct is located behind this bone portion; therefore, a gentle tap should be made on the osteotome to mobilize the fragment of bone after osteotomy in order not to damage the lacrimal duct (Figure 1).

Lateral nasal wall lateralization

**Figure 2:**
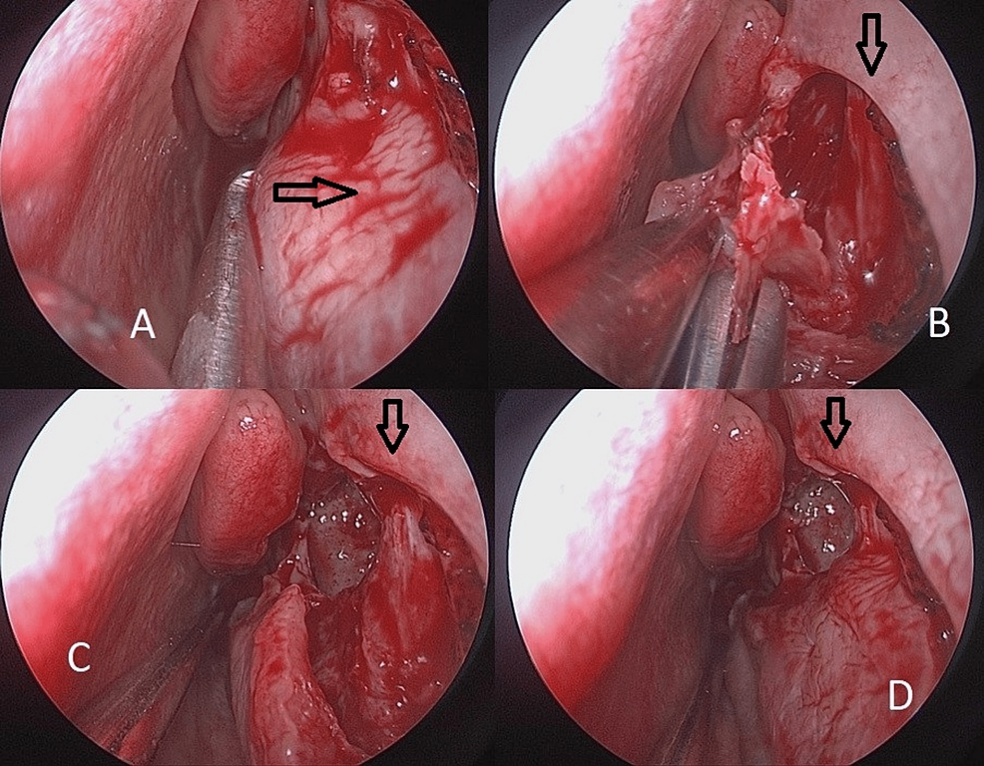
LNWL performed step-by-step. A: Appearance after pyriform turbinoplasty. B: Mucosal flap is raised showing the medial wall of the maxillary sinus. C: Mobilization of the bone to obtain a better view of the maxillary ostia. D: Reposition of the mucosal flap after LNWL, obtaining the final view of the procedure. LNWL = lateral nasal wall lateralization

After PT, one can identify the lacrimal duct. A lateral dissection is made to expose the attachment of the lacrimal bone to the maxilla. The lacrimal bone is then exposed in the posterior, lateral, and inferior directions onto the lacrimal duct. After this gesture, this bone can be now freed: we used a 3 mm osteotome. This bone is present at the junction of the lacrimal bone and uncinate process, lying just behind the lacrimal duct, the base of the IT, and the portion of the maxilla that lies at the pyriform aperture. After this procedure on the lateral nasal wall and the removal of pieces, we obtain a wide nasal cavity while preserving the nasal mucosa (Figure 2).

**Figure 3:**
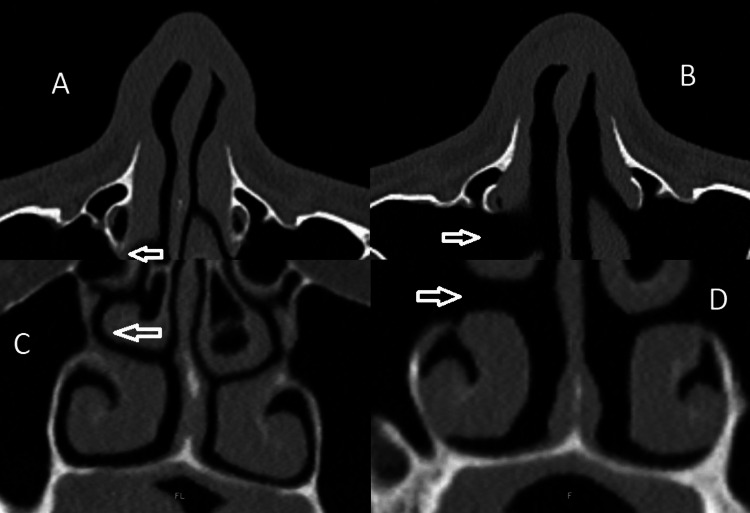
CT scan comparative status before and after PT with LNWL. A: Preoperative view in the axial cut of paranasal sinus CT scan. B: Status after PT and LNWL in the axial cut of paranasal sinus CT scan. C: Preoperative view in the coronal cut of paranasal sinus CT scan. D: Status after PT and LNWL in the coronal cut of paranasal sinus CT scan. CT = computed tomography; PT = pyriform turbinoplasty; LNWL = lateral nasal wall lateralization

PT and LNWL result in a wider nasal cavity, as illustrated by Figures 1-3 and Video 1.

Postoperative management

We advise the use of regular nasal douching with saline solution three to four times a day. This will help with nasal crusting and perioperative temporary nasal obstruction. There is no need for antibiotics after surgery. Pain control is vital as patients experience some discomfort after bony damage. A combination of a non-steroidal anti-inflammatory drug with either acetaminophen or codeine phosphate helps relieve pain.

## Discussion

It is possible to perform PT with LNWL associated with an extended maxillary sinusotomy without damaging the lacrimal duct by removing the bone in this area. It also allows the management of the maxillary sinus with a zero-degree endoscope and major visualization of this region. Care must be taken not to twist the segment of the bone toward the orbit to avoid tearing the periorbita. According to Simmen et al., this technique improved the airflow in the nasal valve, speeding the air that flows into the lungs, thus helping ventilation without altering the normal airflow pattern [2,4]. Any surgical procedure involving the nasal turbinates can lead to a change in nasal air conditioning. It is paradoxical that excessive resection of these structures causes nasal dryness with crusting and a reduction in the feeling of airflow despite an increase in airflow. This technique did not adversely affect the nasal climatization process as the airflow distribution was improved to a more physiological pattern while preserving the turbinate complex [3,4]. Computational fluid dynamics models have been applied to study these processes, comparing those to resections of the ITs, showing that PT and LNWL lowered nasal resistance without influencing nasal climatization [2-4]. Potential complications include bleeding from the lacrimal artery, numbness, given that the area is innervated by the anterior superior alveolar nerve, crusting, and epiphora if the resection compromises the tear sac [5,6]. These complications are not common and are normally short-term in nature. Other techniques such as radiofrequency or ablation of turbinate tissue tend to achieve short-term benefits because of the re-hypertrophy of the mucosa. PT leads to a longer-term benefit as it is directed to the lateral bony margin of the nasal valve [4]. Hypertrophy of ITs plays a major role in nasal airway obstruction [6]. In the management of IT hypertrophy, an approach with the preservation of mucosa is preferred. Even though non-mucosal-saving techniques provide effective relief from nasal obstruction, they have been associated with postoperative complications, including excessive bleeding, crusting, pain, and prolonged recovery. PT with LNWL preserves the mucosa, offering these advantages [7].

## Conclusions

PT improves nasal airflow and provides a wider nasal cavity by acting directly into the bone of the IT. Sustained symptomatic improvement has been documented and is less susceptible to the influence of turbinate hypertrophy that occurs in many other techniques. Further studies are needed to evaluate its morbidity and long-term impact on nasal airflow.

## References

[1] Simmen D, Jones N (2014). Manual of Endoscopic Sinus and Skull Base Surgery.

[2] Simmen D, Sommer F, Briner HR, Jones N, Kröger R, Hoffmann TK, Lindemann J (2015). The effect of "pyriform turbinoplasty" on nasal airflow using a virtual model. Rhinology.

[3] Simmen D, Scherrer JL, Moe K, Heinz B (1999). A dynamic and direct visualization model for the study of nasal airflow. Arch Otolaryngol Head Neck Surg.

[4] Machado A, Briner HR, Schuknecht B, Simmen D (2021). Assessment of the anterior superior alveolar nerve and its impact on surgery of the lateral nasal wall. Rhinology.

[5] Simmen D, Veerasigamani N, Briner HR, Jones N, Schuknecht B (2017). Anterior maxillary wall and lacrimal duct relationship - CT analysis for prelacrimal access to the maxillary sinus. Rhinology.

[6] Courtiss EH (1988). Diagnosis and treatment of nasal airway obstruction due to inferior turbinate hypertrophy. Clin Plast Surg.

[7] Abdullah B, Singh S (2021). Surgical interventions for inferior turbinate hypertrophy: a comprehensive review of current techniques and technologies. Int J Environ Res Public Health.

